# Supplementary material for: Lung cancer symptoms awareness among Ethiopian adults: A latent class analysis
Source: PLoS One. 2025 Oct 23;20(10):e0332952. doi: 10.1371/journal.pone.0332952 (PMC12548894; doi:10.1371/journal.pone.0332952)
Supplement: S1 File — (DOCX) [file pone.0332952.s001.docx]

**Lung cancer symptoms awareness survey tool**

| **SECTION 1: Lung cancer warning signs and symptoms** | | | | | **Code** |
| --- | --- | --- | --- | --- | --- |
| **Recognize warning symptoms**  The following may or may not be warning signs for lung cancer. We are interested in your opinion: | | | | | |
| 101 | Do you think that unexplained weight loss could be a sign of lung cancer? | *Yes* | *No* | *Don’t know* |  |
| 102 | Do you think that a persistent (3 weeks or longer) chest infection could be a sign of lung cancer? | *Yes* | *No* | *Don’t know* |  |
| 103 | Do you think that a cough that does not go away for two or three weeks could be a sign of lung cancer? | *Yes* | *No* | *Don’t know* |  |
| 104 | Do you think that persistent shortness of breath could be a sign of lung cancer? | *Yes* | *No* | *Don’t know* |  |
| 105 | Do you think that persistent tiredness or lack of energy could be a sign of lung cancer? | *Yes* | *No* | *Don’t know* |  |
| 106 | Do you think that persistent chest pain could be a sign of lung cancer? | *Yes* | *No* | *Don’t know* |  |
| 107 | Do you think that persistent shoulder pain could be a sign of lung cancer? | *Yes* | *No* | *Don’t know* |  |
| 108 | Do you think that coughing up blood could be a sign of lung cancer? | *Yes* | *No* | *Don’t know* |  |
| 109 | Do you think that an ache or pain when breathing could be a sign of lung cancer? | *Yes* | *No* | *Don’t know* |  |
| 110 | Do you think that loss of appetite could be a sign of lung cancer? | *Yes* | *No* | *Don’t know* |  |
| 111 | Do you think that a painful cough could be a sign of lung cancer? | *Yes* | *No* | *Don’t know* |  |
| 112 | Do you think that changes in the shape of your fingers or nails could be a sign of lung cancer? | *Yes* | *No* | *Don’t know* |  |
| 113 | Do you think that developing an unexplained loud, high pitched sound when breathing could be a sign of lung cancer? | *Yes* | *No* | *Don’t know* |  |
| 114 | Do you think that worsening or change in an existing cough could be a sign of lung cancer? | *Yes* | *No* | *Don’t know* |  |

| **SECTION 2:1 General information** | | | | | **Code** |  |  |
| --- | --- | --- | --- | --- | --- | --- | --- |
| 201 | Date of birth | | (DD/MM/YYYY) | |  |  |  |
| 202 | Age in years | |  | |  |  |  |
| 203 | Sex | | 1. Male  2. Female | |  |  |  |
| 204 | Marital status | | 1. Single/never married 2. Married 3. Widowed 4. Divorced   77. Other(specify)  88. Don’t know  99. Refuse to respond | |  |  |  |
| 205 | Ethnicity | | 1. Amhara 2. Oromo 3. Tegri   77. Others **(specify)________**  88. don’t know  99. refused to respond | |  |  |  |
| 206 | Religion | | 1. Christian 2. Islam / Muslim   3. Others (specify)_________ | |  |  |  |
| 207 | Highest level of education | | 1. Unable to read and write 2. Able to read and write 3. Primary education 4. Secondary/High school 5. Technical/Vocational 6. University degree & above   **[If the answer is 1 or 2 skips to Q. No-209]** | |  |  |  |
| 208 | Year spend/spent in Education | | _________________years | |  |  |  |
| 209 | Occupation | | 1. Housewife 2. Government employee 3. Private employee 4. Merchant 5. Farmer   77. Others **(specify)_____** | |  |  |  |
| 210 | Average monthly Income | | _____________________Birr | |  |  |  |
| 211 | Have you ever smoked cigarettes? | | 1. Yes, and I still smoke  2. Yes, but I no longer smoke  3. No, I have never smoked | |  |  |  |
| 212 | Average number cigarettes smokes per day | | _____day | |  |  |  |
| 213 | At what age did you start smoking? | | ________Years | |  |  |  |
| 214 | For how many years have you smoked? | | _______years | |  |  |  |
| 215 | Do you know a person with lung cancer? | | 1. No 2. Yes | |  |  |  |
| 216 | If Yes for Q 215 | | 1. You 2. Partner 3. Family member 4. friends   77. Other Specify___________ | |  |  |  |
| 217 | Do you any person with other types of cancer? | | 1. No 2. Yes | |  |  |  |
| 218 | Have you ever been told by a healthcare professional that you have any of the listed medical conditions? | | 1. HTN 2. DM 3. Asthma 4. Bronchitis 5. Cancer   77. Other: Specify_____  88. Don’t know  99. Refused to respond | |  |  |  |
| **Section 2:2 Socio economic and other characteristics of participants** | | | | | | | |
| **Q. No** | | | **Question** | | **Possible answers and coding** | | |
| **301** | | | **\| \|**Does your household have | | 1. Electricity  2. Watch/clock  3. Radio  4. Television  5. Mobile telephone  6. Non-mobile telephone  7. Refrigerator  8. Table  9. Chair  10. Bed  11. Electric Mittad  12. Kerosene lamp **[If no, go to 302]** | | |
| **302** | | | **\| \|**What type of fuel does your household mainly use for cooking? | | 1. Electricity 2. Natural gas  3. Biogas 4. Charcoal  5. Wood 6. Straw/shrub/grass  7. Agriculture crop 8. Animal dung  77. Other (specify): __________ | | |
| **303** | | | **\| \|**Main material of the floor of your house | | 1. Earth sand  2. Dung  3. wood planks  4. palm/bamboo  5. cement  6. carpe  77. Other (specify) _________ | | |
| **304** | | | **\| \|**Main material of the roof of your house | | 1. Thatch/leaf/mud  2. Corrugated iron /metal  3. Rustic mat/plastic sheets  4. Reed/bamboo  5. Cardboard  6. Wood  77. Other (specify)___ | | |
| **305** | | | **\| \|**Main material of the exterior wall of your house | | 1.No walls  2. Cane/trunks/bamboo/reed  3. Bamboo/wood with mud  4. Stone with mud  5. Stone with lime/cement  6. Wood planks/shingles  77. Other (specify)______ | | |
| **306** | | | **\| \|**Home ownership | | 1.Private 2. Rent  3. Government 77. Other specify______ | | |
| **307** | | | **\| \|**How many rooms is available in the house for sleeping? | | ____________No rooms | | |
| **308** | | | **\| \|**Do you have any agricultural land | | 1-No 2-Yes_______ in Hectare | | |
| **309** | | | **\| \|**Do you have any livestock, herds, other farm animals, or poultry | | 1-No 2-Yes  **[If No skip to Q. No-311]** | | |
| **310** | | | **\| \|**How many of the following animals do your household own? **If none, record '00'. If 95 or more, record '95'. If unknown, record '88'.** | | 1-Cows____  2-Oxen or Bulls____  3-Horses/Mule___  4-Goats____  5-Sheep____  6-Chickens or other poultry__  7-Beehives___  8-Donkeys_____ | | |
| **311** | | | **\| \|**Does any member of this household have a bank account? | | 1-No 2-Yes | | |
| **312** | | | **\| \|**How do you usually pay for medical care? | | 1. Out of pocket  2. Community based health insurance  3. Family and partner support  77. Other, specify_____________ | | |

**END of the questionnaire**: Thank you very much for the time you have given us!

**About the interview**

**[001]** Name of interviewer: ...................................................................................

**[002]** Date of interview completed (dd/mm/yyyy) | | |/| | |/| | | | |

**[003]** Time of finishing the interview: | | | : | | |

(12 hr format) Circle if A.M. or P.M.

**[004]** any additional comments about this interview [text]:
