# Supplementary material for: Lung cancer symptoms awareness among Ethiopian adults: A latent class analysis
Source: PLoS One. 2025 Oct 23;20(10):e0332952. doi: 10.1371/journal.pone.0332952 (PMC12548894; doi:10.1371/journal.pone.0332952)
Supplement: S1 Checklist — (DOCX) [file pone.0332952.s002.docx]

STROBE Statement—checklist of items that should be included in reports of observational studies

|  | Item No. | Recommendation | Page  No. | Relevant text from the manuscript |
| --- | --- | --- | --- | --- |
| **Title and abstract** | 1 | (*a*) Indicate the study’s design with a commonly used term in the title or the abstract | 2 | It is indicated by the word “A population-based cross-sectional survey” in the abstract |
|  |  | (*b*) Provide in the abstract an informative and balanced summary of what was done and what was found | 2 | The abstract methods and result section indicated what was done & what was found. |
| Introduction | | | |  |
| Background/rationale | 2 | Explain the scientific background and rationale for the investigation being reported | 3-4 | The introduction section of the manuscript presents the rationale and existing literature. |
| Objectives | 3 | State specific objectives, including any prespecified hypotheses | 4 | The last paragraph of the introduction section  “the primary objectives of the study were to identify subgroups of the population according to their level of lung cancer symptoms awareness and predictors of class membership using LCA” **Page 4, line number 112-117** |
| Methods | | | |  |
| Study design | 4 | Present key elements of study design early in the paper | 5-11 | The study design is indicated as “Population-based cross-sectional survey” **Page 5, line number 121**  All key elements of the study design are included in the method section of the manuscript. |
| Setting | 5 | Describe the setting, locations, and relevant dates, including periods of recruitment, exposure, follow-up, and data collection | 5, 6, 7, 8 | The setting, locations, and relevant dates, including periods of recruitment, exposure, and data collection are included in the method section. **follow-up does not apply as the study was a population-based cross-sectional survey.** |
| Participants | 6 | (*a*) *Cohort study*—Give the eligibility criteria and the sources and methods of selection of participants. Describe methods of follow-up  *Case-control study*—Give the eligibility criteria and the sources and methods of case ascertainment and control selection. Give the rationale for the choice of cases and controls  ***Cross-sectional study***—Give the eligibility criteria and the sources and methods of selection of participants | 5 -6 | Study population is described in the method section (Study population headline), as well as selection criteria “The source population for this study included all adults residing in Addis Ababa, and the study population consisted of randomly selected adults. Only adults who were 18 years of age or older and had been residing in Addis Ababa for a minimum of 6 months were eligible to participate. However, individuals with mental illness or severe illness were not included in the study” **Page 5, line number 132-135**  The method of selection is described under sample size determination and sampling procedure section “Participants for the study were enrolled using a multistage stratified cluster sampling method. The sampling frame included a comprehensive list of all census enumeration areas in Addis Ababa as of 2019, prepared by the central statistical agency for the Population and Housing Census. From this list, 96 enumerator areas were randomly selected from the eleven sub-cities of Addis Ababa, considering their sizes. Then, 25 households were selected from each enumerator area using systematic random sampling. Within each chosen household, one eligible adult was interviewed. If there were multiple eligible adults in a selected household, the lottery method was used to choose one. Any adults who declined to participate or could not be reached after adults from neighbouring households did not replace multiple attempts.” **Page 5-6, line number 146-154** |
|  |  | (*b*) *Cohort study*—For matched studies, give matching criteria and number of exposed and unexposed  *Case-control study*—For matched studies, give matching criteria and the number of controls per case |  |  |
| Variables | 7 | Clearly define all outcomes, exposures, predictors, potential confounders, and effect modifiers. Give diagnostic criteria, if applicable | 6-7 | Outcome and exposure variables have been adequately described. |
| Data sources/ measurement | 8* | For each variable of interest, give sources of data and details of methods of assessment (measurement). Describe comparability of assessment methods if there is more than one group | *6-8* | All variables/data were collected from participants’ self-reports except the wealth report, which is created based on observable household features and possessions. |
| Bias | 9 | Describe any efforts to address potential sources of bias | 6- 11 | Efforts to address potential sources of bias are described in the Methods subsection data collection tools and measurement (**Pages 6 & 7),** quality assurance (**Page 8, line number 222-233**), and data processing and analysis (**Pages 8-11)** |
| Study size | 10 | Explain how the study size was arrived at | 5 | The sample size determination is indicated on **Page 5, lines 136-145** |

Continued on next page

| Quantitative variables | 11 | Explain how quantitative variables were handled in the analyses. If applicable, describe which groupings were chosen and why | 6-8 | The method section subsection Data Collection Tools and Measurement provides a detailed explanation of how the variables were grouped and defined. In this process, we considered both theoretical and statistical criteria to ensure a comprehensive approach. |
| --- | --- | --- | --- | --- |
| Statistical methods | 12 | (*a*) Describe all statistical methods, including those used to control for confounding | 8-10 | The methods of analysis have been elaborated extensively.  An appropriate regression model was employed to account for confounding variables. |
|  |  | (*b*) Describe any methods used to examine subgroups and interactions | N/A | Does not apply. Our models do not incorporate any type of interaction. |
|  |  | (*c*) Explain how missing data were addressed | N/A | We have no missing data |
|  |  | (*d*) *Cohort study*—If applicable, explain how loss to follow-up was addressed  *Case-control study*—If applicable, explain how matching of cases and controls was addressed  *Cross-sectional study*—If applicable, describe analytical methods taking account of sampling strategy | 10 | We have clarified our analytical techniques, which thoroughly consider the sampling approach. **Page 10 & 11, line number 299-303** |
|  |  | (*e*) Describe any sensitivity analyses | N/A | We did not do sensitivity analyses |
| Results | | | | |
| Participants | 13* | (a) Report numbers of individuals at each stage of study—eg numbers potentially eligible, examined for eligibility, confirmed eligible, included in the study, completing follow-up, and analysed | 11 | This is described at the beginning of the result section “Out of the total of 2,388 adults invited for the study, 2,341 adults participated, resulting in a response rate of 98%.” **Page 11, line number 317-318** |
|  |  | (b) Give reasons for non-participation at each stage | 11 | Non-participation happened only at individual level this is described at the beginning of the result section “Out of the total of 2,388 adults invited for the study, 2,341 adults participated, resulting in a response rate of 98%.” **Page 11, line number 317-318** |
|  |  | (c) Consider use of a flow diagram | NA | The utilization of a flow diagram was considered unsuitable |
| Descriptive data | 14* | (a) Give characteristics of study participants (eg demographic, clinical, social) and information on exposures and potential confounders | 11-13 | The information has been presented in Table 1 and Table 2. |
|  |  | (b) Indicate number of participants with missing data for each variable of interest | N/A | Our dataset does not contain any missing data. |
|  |  | (c) *Cohort study*—Summarise follow-up time (eg, average and total amount) |  |  |
| Outcome data | 15* | *Cohort study*—Report numbers of outcome events or summary measures over time |  |  |
|  |  | *Case-control study—*Report numbers in each exposure category, or summary measures of exposure |  |  |
|  |  | *Cross-sectional study—*Report numbers of outcome events or summary measure | *11-20* | The summary measures were presented in tables, figures, and texts primarily within the result section. |
| Main results | 16 | (*a*) Give unadjusted estimates and, if applicable, confounder-adjusted estimates and their precision (eg, 95% confidence interval). Make clear which confounders were adjusted for and why they were included | 18-19 | Table 4 presents the weighted adjusted estimates along with their corresponding 95% confidence intervals. Additionally, the unweighted adjusted estimate is included as a supplementary document. |
|  |  | (*b*) Report category boundaries when continuous variables were categorized | 12 | Table 1 presents the category boundaries for age groups, while all the remaining variables were already categorical. |
|  |  | (*c*) If relevant, consider translating estimates of relative risk into absolute risk for a meaningful time period | N/A | N/A |

Continued on next page

| Other analyses | 17 | Report other analyses done—eg analyses of subgroups and interactions, and sensitivity analyses | N/A | N/A |
| --- | --- | --- | --- | --- |
| Discussion | | | | |
| Key results | 18 | Summarise key results with reference to study objectives | 20 | The opening paragraphs of the discussion have provided a comprehensive description of it |
| Limitations | 19 | Discuss limitations of the study, taking into account sources of potential bias or imprecision. Discuss both direction and magnitude of any potential bias | 25 | In the final section of the discussion, we have outlined this information on **Page 25, specifically in lines 635-637.** |
| Interpretation | 20 | Give a cautious overall interpretation of results considering objectives, limitations, multiplicity of analyses, results from similar studies, and other relevant evidence | 20-25 | Citations were included wherever applicable and deliberated upon. Limitation were acknowledged in the discussion. |
| Generalisability | 21 | Discuss the generalisability (external validity) of the study results | 20-26 | The findings of the study were considered applicable to the broader Ethiopian context and were deliberated in that light. |
| Other information | |  | | |
| Funding | 22 | Give the source of funding and the role of the funders for the present study and, if applicable, for the original study on which the present article is based |  | The funding details were shown during the submission process but were not incorporated into the manuscript, as per the request. |

*Give information separately for cases and controls in case-control studies and, if applicable, for exposed and unexposed groups in cohort and cross-sectional studies.

**Note:** An Explanation and Elaboration article discusses each checklist item and gives methodological background and published examples of transparent reporting. The STROBE checklist is best used in conjunction with this article (freely available on the Web sites of PLoS Medicine at http://www.plosmedicine.org/, Annals of Internal Medicine at http://www.annals.org/, and Epidemiology at http://www.epidem.com/). Information on the STROBE Initiative is available at www.strobe-statement.org.
