# Supplementary material for: Lung cancer symptoms awareness among Ethiopian adults: A latent class analysis
Source: PLoS One. 2025 Oct 23;20(10):e0332952. doi: 10.1371/journal.pone.0332952 (PMC12548894; doi:10.1371/journal.pone.0332952)
Supplement: S1 Table — (DOCX) [file pone.0332952.s003.docx]

**S1 Table.** Weighted latent class marginal means of lung cancer symptoms awareness survey in Addis Ababa, Ethiopia ,2023

|  |  |  |  |
| --- | --- | --- | --- |
| **Manifest variables** | **Class 1** | **Class 2** | **Class 3** |
| **Class share** | 0.379 | 0.375 | 0.246 |
| Unexplained weight loss | 0.135 | 0.677 | 0.903 |
| A persistent (3 weeks or longer) chest infection | 0.156 | 0.750 | 0.948 |
| A cough that does not go away for two or three weeks | 0.332 | 0.911 | 0.981 |
| Persistent shortness of breath | 0.155 | 0.878 | 0.978 |
| Persistent tiredness or lack of energy | 0.149 | 0.843 | 0.991 |
| Persistent chest pain | 0.070 | 0.693 | 0.994 |
| Persistent shoulder pain | 0.024 | 0.235 | 0.721 |
| Coughing up blood | 0.345 | 0.934 | 0.967 |
| An ache or pain when breathing | 0.098 | 0.857 | 0.988 |
| Loss of appetite | 0.122 | 0.725 | 0.986 |
| A painful cough | 0.234 | 0.912 | 1 |
| Changes in the shape of your fingers or nails | 0.033 | 0.143 | 0.541 |
| Developing an unexplained loud, high-pitched sound when breathing | 0.050 | 0.578 | 0.983 |
| Worsening or change in an existing cough | 0.254 | 0.885 | 0.994 |

**S1 Table**. Unweighted Latent class marginal means of lung cancer symptoms awareness survey in Addis Ababa, Ethiopia ,2023

|  |  |  |  |
| --- | --- | --- | --- |
| **Manifest variables** | **Class 1** | **Class 2** | **Class 3** |
| **Class share** | 0.395 | 0.373 | 0.232 |
| Unexplained weight loss | 0.141 | 0.691 | 0.921 |
| A persistent (3 weeks or longer) chest infection | 0.165 | 0.756 | 0.924 |
| A cough that does not go away for two or three weeks | 0.315 | 0.912 | 0.972 |
| Persistent shortness of breath | 0.154 | 0.854 | 0.982 |
| Persistent tiredness or lack of energy | 0.134 | 0.820 | 0.998 |
| Persistent chest pain | 0.071 | 0.704 | 0.990 |
| Persistent shoulder pain | 0.022 | 0.265 | 0.729 |
| Coughing up blood | 0.335 | 0.937 | 0.970 |
| An ache or pain when breathing | 0.099 | 0.841 | 0.985 |
| Loss of appetite | 0.121 | 0.749 | 0.989 |
| A painful cough | 0.241 | 0.910 | 1 |
| Changes in the shape of your fingers or nails | 0.041 | 0.143 | 0.536 |
| Developing an unexplained loud, high-pitched sound when breathing | 0.053 | 0.574 | 0.975 |
| Worsening or change in an existing cough | 0.246 | 0.881 | 0.998 |
