# Supplementary material for: Lung cancer symptoms awareness among Ethiopian adults: A latent class analysis
Source: PLoS One. 2025 Oct 23;20(10):e0332952. doi: 10.1371/journal.pone.0332952 (PMC12548894; doi:10.1371/journal.pone.0332952)
Supplement: S2 Table — (DOCX) [file pone.0332952.s004.docx]

**S2_Table: Unweighted Latent class multinomial logistic regression of predictors of latent class membership Addis Ababa, Ethiopia,2023**

|  | Class 2: “fair awareness class” **^1^** |  | Class 3: “good awareness class” **^1^** |  |
| --- | --- | --- | --- | --- |
| **Variables ^1^** | AOR (95 % CI) | P-Value | AOR (95 % CI) | P-Value |
| **Sex** |  |  |  |  |
| Male | 1.22 (0.94, 1.58) | 0.145 | 1.49 (1.08, 2.05) | **0.016** |
| Female | 1 |  |  |  |
| **Age (years)** |  |  |  |  |
| 30-39 | 0.92 (0.71, 1.23) | 0.608 | 1.07(0.76, 1.58) | 0.692 |
| 40-49 | 0.81 (0.58, 1.12) | 0.199 | 0.95 (0.63, 1.43) | 0.796 |
| 50-59 | 1.30 (0.86, 1.95) | 0.217 | 1.27(0.74, 2.17) | 0.382 |
| >60 | 0.81 (0.51, 1.28) | 0.363 | 0.86(0.44, 1.66) | 0.65 |
| 18-29 | 1 |  | 1 |  |
| **Level of education** |  |  |  |  |
| Able to read and write | 0.74 (0.44, 1.25) | 0.253 | 0.53 (0.15, 2.16) | 0.333 |
| Primary education | 0.96 (0.59, 1.56) | 0.867 | 1.93 (0.69, 5.37) | 0.211 |
| Secondary education | 1.44 (0.884, 2.34) | 0.143 | 3.38 (1.22, 9.35) | **0.019** |
| Diploma and vocational | 1.63 (0.96, 2.78) | 0.073 | 4.82 (1.71, 13.7) | **0.003** |
| Degree and above | 2.19 (1.252, 3.812) | **0.006** | 7.90 (2.75, 22.9) | **0.000** |
| Unable to read and write | 1 |  | 1 |  |
| **Occupation** |  |  |  |  |
| Employee | 1.34 (0.89, 2.00) | 0.157 | 1.63 (1.02, 2.61) | **0.041** |
| Merchant | 1.04(0.78, 1.38) | 0.806 | 0.78 (0.53, 1.15) | 0.214 |
| Student | 0.94 (0.54, 1.63) | 0.814 | 0.90 (0.45, 1.79) | 0.752 |
| Unemployed | 1.44 (0.75, 2.75) | 0.276 | 1.05 (0.47, 2.45) | 0.902 |
| Others | 1.19 (0.62, 2.27) | 0.602 | 0.49 (0.17, 1.42) | 0.189 |
| House wife | 1 |  | 1 |  |
| **Wealth index** |  |  |  |  |
| Poorer | 1.29 (0.95, 1.77) | 0.109 | 1.08 (0.70, 1.66) | 0.726 |
| Medium | 1.30 (0.91, 1.85) | 0.146 | 1.62 (1.05, 2.50) | **0.029** |
| Richer | 1.97 (1.37, 2.82) | **0.000** | 2.19 (1.34, 3.58) | **0.001** |
| Richest | 1.74 (1.28, 2.36) | **0.000** | 1.48 (0.98,2.25) | 0.066 |
| Poorest | 1 |  |  |  |
| **Payment option for medical services** |  |  |  |  |
| Family and partner support | 0.47 (0.19, 1.15) | 0.098 | 0.77 (0.23, 2.54) | 0.664 |
| Out pocket money | 1.37 (1.07, 1.75) | **0.011** | 2.10 (1.47, 3.00) | **0.000** |
| Others | 2.96 (0.90, 9.76) | 0.074 | 1.96 (0.47, 8.12) | 0.355 |
| CBHI | 1 |  |  |  |
| **Ever smoke cigarette** |  |  |  |  |
| Yes | 1.22 (0.77, 1.93) | 0.407 | 1.34 (0.79, 2.26) | 0.278 |
| No | 1 |  |  |  |
| **Have chronic diseases** |  |  |  |  |
| Yes | 0.86 (0.65, 1.15) | 0.303 | 0.99 (0.69, 1.43) | 0.948 |
| No | 1 |  |  |  |
| **Know person with lung cancer** |  |  |  |  |
| Yes | 1.584(0.62, 4.08) | 0.341 | 3.97 (1.59, 9.11) | **0.003** |
| No | 1 |  |  |  |
| **Know person with other cancer** |  |  |  |  |
| Yes | 1.41 (0.97, 2.04) | **0.040** | 2.66 (1.81, 3.90) | **0.000** |
| No | 1 |  | 1 |  |
